# Pustulan Activates Chicken Bone Marrow-Derived Dendritic Cells In Vitro and Promotes Ex Vivo CD4^+^ T Cell Recall Response to Infectious Bronchitis Virus

**DOI:** 10.3390/vaccines8020226

**Published:** 2020-05-15

**Authors:** Frederik T. Larsen, Bernt Guldbrandtsen, Dennis Christensen, Jacob Pitcovski, Rikke B. Kjærup, Tina S. Dalgaard

**Affiliations:** 1 Department of Animal Science, Aarhus University, Blichers Allé 20, 8830 Tjele, Denmark; rikke.kjaerup@anis.au.dk; 2 Center for Quantitative Genetics and Genomics, Blichers Allé 20, 8830 Tjele, Denmark; bernt.guldbrandtsen@mbg.au.dk; 3 Department of Infectious Disease Immunology, Statens Serum Institut, Artillerivej 5, DK-2300 Copenhagen S, Denmark; den@ssi.dk; 4 Virology and Vaccine Development Laboratory, MIGAL Technology Center, Kiryat Shmona 11016, Israel; jp@migal.org.il

**Keywords:** chicken, pustulan, BM-DC, IBV, subunit vaccination, adjuvant, APC-targeting

## Abstract

Infectious bronchitis virus (IBV) is a highly contagious avian coronavirus. IBV causes substantial worldwide economic losses in the poultry industry. Vaccination with live-attenuated viral vaccines, therefore, are of critical importance. Live-attenuated viral vaccines, however, exhibit the potential for reversion to virulence and recombination with virulent field strains. Therefore, alternatives such as subunit vaccines are needed together with the identification of suitable adjuvants, as subunit vaccines are less immunogenic than live-attenuated vaccines. Several glycan-based adjuvants directly targeting mammalian C-type lectin receptors were assessed in vitro using chicken bone marrow-derived dendritic cells (BM-DCs). The β-1-6-glucan, pustulan, induced an up-regulation of MHC class II (MHCII) cell surface expression, potentiated a strong proinflammatory cytokine response, and increased endocytosis in a cation-dependent manner. Ex vivo co-culture of peripheral blood monocytes from IBV-immunised chickens, and BM-DCs pulsed with pustulan-adjuvanted recombinant IBV N protein (rN), induced a strong recall response. Pustulan-adjuvanted rN induced a significantly higher CD4^+^ blast percentage compared to either rN, pustulan or media. However, the CD8^+^ and TCRγδ^+^ blast percentage were significantly lower with pustulan-adjuvanted rN compared to pustulan or media. Thus, pustulan enhanced the efficacy of MHCII antigen presentation, but apparently not the cross-presentation on MHCI. In conclusion, we found an immunopotentiating effect of pustulan in vitro using chicken BM-DCs. Thus, future in vivo studies might show pustulan as a promising glycan-based adjuvant for use in the poultry industry to contain the spread of *coronaviridiae* as well as of other avian viral pathogens.

## 1. Introduction

Outbreaks of infection caused by coronaviridiae, such as infectious bronchitis virus (IBV), cause substantial economic losses in the poultry industry worldwide. Vaccination programs, therefore, are of critical importance to prevent outbreaks of IBV and other avian diseases. Live-attenuated viral vaccines have good immunogenicity and are commonly used to induce protection against IBV [1]. The activation of the humoral immune system has been shown to be important for IBV-vaccine-induced protection using live-attenuated viral vaccines [2]. However, the correlation between serum anti-IBV titres and protection is lacking [3,4,5]. Moreover, live-attenuated viral vaccines exhibit the potential for reversion to virulence as well as the recombination with virulent field strains with the outcome of a new variant [6]. This makes it desirable to find alternatives such as subunit or inactivated viral vaccines. In addition, new variants appear relatively frequently and the adaptation of an attenuated vaccine is a long process whereas the adjustment of a subunit vaccine is relatively fast. However, subunit- or inactivated viral-based vaccines are far less immunogenic compared to live-attenuated vaccines. Thus, immunopotentiating agents (adjuvants) are needed to boost vaccine efficacy [7,8]. Adjuvants directly targeting professional antigen-presenting cells (APCs) trigger the innate immune system, which can lead to an effective pathogen-specific adaptive immune response [9,10,11].

The binding and subsequent phagocytosis of pathogens by APCs is mediated by pattern-recognition receptors (PRR). PRRs bind evolutionarily conserved extra- and intracellular pathogen-associated molecular patterns (PAMPs). Dendritic cells (DCs) are key APCs as they are potent activators of naive T cells. In mammals, the binding of a PAMP by its cognate PRR activates APCs leading to APC maturation. The process of APC maturation entails the secretion of proinflammatory cytokines, an increase in MHC class II (MHCII) cell surface expression, chemokine receptors and co-stimulatory molecules and subsequent migration to secondary lymphoid tissues for antigen presentation to naive T cells. This in turn leads to the activation of the adaptive immune system. PRR-mediated endocytosis of pathogens by APCs triggers a pathogen-specific cytokine response leading to the differentiation of different classes of T-helper cells such as T-helper 1 (Th1) or T-helper 2 (Th2) cells. Targeting APCs using PAMPs can thereby efficiently prime the adaptive immune system towards a specific pathogen. Hence, APC-targeting using PAMPs has gained increasing interest in adjuvant development [12] as the targeting of PRRs expressed by APCs can create a pathogen-specific adaptive immune response.

Several PRRs have been well characterised in mammals. These include Toll-like receptors (TLRs) [13,14], nucleotide-binding oligomerization domain (NOD) -like receptors [14], retinoic acid-inducible gene-I (RIG-I) -like receptors [15], cytosolic DNA sensors [16] and C-type lectin receptors (CLRs) [17]. CLRs bind glycans in a Ca^2+^-dependent manner and share one or more common carbohydrate-recognition domains, which contain highly conserved amino acid motifs critical for tertiary structure and the Ca^2+^ coordination implicated in glycan binding [18].

In recent years, CLRs expressed by APCs have received growing interest for APC targeting as CLRs facilitate the internalisation of antigens and can be selectively targeted using antibodies or glycan-based ligands [19]. The targeting of human and mouse CLRs by monoclonal antibodies (mAbs) or single chain variable fragments (scFv) conjugated to antigens has become the preferred strategy [20]. Glycan-based ligands, however, present some advantages over mAbs as they are biodegradable, exhibit low intrinsic immunogenicity [19,21] and can be designed to adjust their spatial orientation and increase their valence [19,22]. The production costs of glycan-based ligands are low compared to mAbs, which renders the utilisation of glycan-based ligands in APC-targeting vaccine designs feasible. In mammals, glycan-based ligands have been shown to be potent adjuvants when coupled to antigens, as they improve antigen uptake, presentation and the subsequent activation of antigen-specific CD4^+^ and CD8^+^ T cells [23,24,25,26].

In chickens, targeting CLRs for antigen delivery to the APCs has been demonstrated using recombinant hemagglutinin (rHA) conjugated to a mAb [27] or a scFv [28] directed against chicken DEC205. Moreover, a study by de Geus et al. [29] showed the mitigated bone marrow-derived dendritic cell (BM-DC) binding of highly pathogenic avian influenza virus (AIV) H7N1, when the BM-DCs were pre-incubated with N-acetylglucosamine (GlcNAc) or N-acetylgalactosamine (GalNAc). This suggests the involvement of one or more chicken CLRs in the binding of AIV H7N1, which recognise terminal GlcNAc or GalNAc moieties. Thus, as in mammals, targeting antigens to chicken APCs using mAbs, scFv or glycan-based CLR agonists seems feasible, which makes glycan-based ligands promising adjuvants to boost inactivated or subunit chicken vaccines.

The potent immunostimulatory effects of mammalian CLR-specific glycan-based ligands pustulan, mannan, chitosan, furfurman, monomycolyl glycerol (MMG), and Trehalose-6,6-dibehenate (TDB) are known [30,31,32,33,34]. However, the recognition of these glycan-based ligands by chicken CLRs and their immunostimulatory potential are not well described for chicken cells. This currently limits the application of these as adjuvants in the vaccination of chicken. The aim of this study, therefore, was to apply an in vitro model using chicken BM-DCs to investigate the immunostimulatory effect of several glycan-based CLR ligands in order to identify glycan-based adjuvants, which may potentially boost the efficacy in vivo of subunit and inactivated avian viral vaccines.

## 2. Materials and Methods

### 2.1. Animals and Ethics

All the chickens used in this study were from the inbred L21 AU/DIAS line [1,2] of the B21 MHC haplotype. For the generation of BM-DCs, the chickens were kept non-vaccinated and used at 3–8 weeks of age. Adult IBV-immunised chickens were used as peripheral blood mononuclear cell (PBMC) donors. These chickens were previously vaccinated mucosally with one dose of live-attenuated Nobilis IB Ma5 Vet vaccine (MSD Animal Health) at 1–4 weeks of age and received a booster vaccination with one dose 4 weeks after the first vaccination (one dose > 10^3.5^ EID_50_). Heparin-stabilised blood from the IBV-immunised chickens at 10–11 months of age was collected for PBMC purification in BD Vacutainer^®^ Blood Collection Plasma Tubes coated with 60 USP Units of Sodium Heparin (BD, Franklin Lakes, NJ, USA).

The MHC types of all the chickens were verified by a fragment analysis of the LEI258 microsatellite locus [35] as previously described [36]. In brief, genomic DNA was isolated from the peripheral blood using the NucleoSpin^®^ 96 Blood core Kit (Macherey-Nagel, Düren, Germany). PureTaq Ready-To-Go PCR beads (GE Healthcare, Uppsala, Sweden) were used for PCR with forward primer Lei258A (5′-ACGCAGCAGAACTTGGTAAGG-3′) and reverse primer Lei258B (5′-AGCTGTGCTCAGTCCTCAGTGC-3′) as described by McConnell et al., 1999 [35]. Amplification by PCR and visualisation by gel electrophoresis were performed as previously described (Dalgaard et al., 2005) [36].

All animals were kept according to the protocols approved by the Danish Animal Experiments Inspectorate and complied with the Danish Ministry of Justice Law no. 382 (10 June 1987) and Acts 739 (6 December 1988) and 333 (19 May 1990) concerning animal experimentation and care of experimental animals, licence holder Dr. med. vet. Ricarda Engberg (Licence no. 2017-15-0201-01211).

### 2.2. Cell Culture

The femurs and tibias of MHC-B line B21 chickens were collected from 3 to 8 week of age and stored in sterile phosphate-buffered saline (PBS) (Lonza, cat. no.: BE17-516F) on ice. In aseptic conditions, the muscle was removed from the bones and the bone marrow was flushed with PBS using a 21G needle. The cells were pelleted (400× *g*, 10 min) and the cell pellet was resuspended in 10 mL pre-warmed RPMI-1640 (Gibco™, cat. no. 21870084). Subsequently, the cells were carefully overlaid an equal volume of Histopaque 1.077 (Sigma-Aldrich, St. Louis, MS, USA) and centrifuged (400× *g*, 20 min, brakes off). The interphase was collected, resuspended in 30 mL pre-warmed RPMI-1640 and the cells pelleted (400× *g*, 10 min). The pelleted cells were resuspended in 10 mL pre-warmed RPMI-1640. The cells were seeded in 6-well plates at 3.125 × 10^5^ cells/cm^2^ in RPMI-1640 supplemented with 2 mM L-Glutamine (Fisher Scientific, Hampton, UK), 0.1% penicillin and streptomycin (Pen–Strep) (Gibco™), and 10 % FBS (Gibco). For the derivation of BM-DCs, recombinant chicken IL-4 (Kingfisher Biotech Inc., St Paul, MN, USA) and recombinant chicken GM-CSF ((Kingfisher Biotech Inc.) were added to the culture medium at a working concentration of 10 ng/mL each. The cells were incubated at 41 °C in a 5% CO_2_ atmosphere for 4 days to allow for BM-DC differentiation.

### 2.3. Glycan-Based Ligand Stimulation of BM-DCs

After 4 days of derivation, the BM-DCs were stimulated for 1, 3 and 6 h with the following (*n* = 3 wells/treatment): RPMI-1640, 2.5 doses/well (equivalent to at least 2.5 × 10^3.5^ EID_50_) UV-inactivated IBV (Nobilis IB Ma5 Vet vaccine, MSD Animal Health), 100 µg/mL pustulan from *Lasallia pustulata* (InvivoGen, San Diego, CA, USA), 500 µg/mL Mannan from *Saccharomyces cerevisiae* (Sigma-Aldrich), 200 µg/mL chitosan (Sigma-Aldrich, cat. no. C3646), 10 µg/mL furfurman from *Malassezia furfur* (InvivoGen, cat. no. tlrl-ffm), 50 µg/mL MMG, or 50 µg/mL TDB. MMG and TDB were provided by Statens Serum Institut. Post stimulation, the BM-DCs were washed 3 times in ice-cold PBS, incubated in 10 mM EDTA on ice for 10 min and detached by thoroughly pipetting up and down. Subsequently, the cells were analysed by flow cytometry. The level of endotoxin contamination was determined by providers or previous studies to be insignificant in the glycan-based ligand concentrations used [34,37,38].

### 2.4. Flow Cytometry

The cells were washed in PBS, pelleted by centrifugation (400× *g*, 10 min) and resuspended in PBS. The cells were added into 96-well U-bottom plates (Falcon) and mixed 1:1 with the following antibody solutions:

BM-DC activation panel was used to identify CD45^+^MHCII^+^MRC1L-B^+^ BM-DCs and contained anti-CD45-PerCP-Cy5.5 (clone UM16-6), anti-MHCII-PE (clone 2G11), anti-monocyte/macrophage-A647 (MRC1L-B/KUL-01) (clone KUL01) and LIVE/DEAD™ Fixable Aqua Dead Cell Stain (Invitrogen™, Carlsbad, CA, USA, cat. no. L34957) for the exclusion of non-viable cells.

The ex vivo blast transformation panel was used to identify blast transformed CD4^+^, CD8^+^ and TCRγδ^+^ T cells and contained anti-CD4-pBlue (clone CT-4), anti-TCR1-PE (clone TCR1), anti-CD8α-Cy5 (clone 3–298), and LIVE/DEAD™ Fixable Near-IR Dead Cell Stain (Invitrogen™, cat. no. L10119) to exclude non-viable cells.

BM-DC activation and ex vivo blast transformation panel staining was performed for 15 min at 4 °C and room temperature, respectively. The PBMCs were washed twice in PBS (600× *g*, 5 min) and resuspended in PBS. Monoclonal antibody anti-CD45-PerCP-Cy5.5 was obtained from Bio-Rad laboratories (Kidlington, UK) and the remaining were obtained from Southern Biotech (Birmingham, AL, USA). Lightning Link from Innova Bioscience was used for the conjugation of anti-CD45 with PerCP-Cy5.5. Each antibody was titrated prior to analysis to determine the optimal concentration for staining.

The flow cytometric analyses were conducted on a BD FACSCelesta^TM^ flow cytometer. Of each sample, 50 µL was acquired and recorded at a flow rate of 1 µL/sec for the PBMCs and 0.5 µL/sec for the BM-DCs. The analyses of the acquired samples were performed with the FACSDiva software.

To correct the gating for both panels, background fluorescence in each fluorescence channel was determined by including an unstained control, single stained controls and negative fluorescence minus one (FMO) controls.

### 2.5. Endocytosis Assay

The BM-DC cells were prepared and seeded as described above in 6-well plates for the analysis of endocytosis. FITC-BSA (10 µg/mL) (Invitrogen™, cat. no. A23015) was mixed with and without 10 mM EDTA and medium control, pustulan (100 µg/mL), mannan (500 µg/mL) or chitosan (200 µg/mL), then added to the BM-DCs (*n* = 3/treatment). Endocytosis was carried out for 1 h at 41 °C or 4 °C (negative control). The BM-DCs were washed 3 times in ice-cold PBS and detached using 10 mM EDTA on ice for 10 min and thoroughly pipetting up and down. Detached BM-DCs were pelleted in ice-cold PBS (600× *g*, 10 min), resuspended in PBS and subsequently stained as described above with LIVE/DEAD™ Fixable Near-IR Dead Cell Stain to exclude non-viable cells. Flow cytometric analysis was conducted as described above to analyse the FITC-positive BM-DCs.

### 2.6. Confocal Microscopy

The intracellular localisation of the endocytosed FITC-labelled BSA was verified by confocal microscopy. The BM-DCs were seeded on collagen-coated coverslips (VWR, Radnor, PA, USA, cat. no. 631-0150) in 12-well plates at a density of 3.125 × 10^5^ cells/cm^2^ and treated as described above. The BM-DCs were stained with fresh CellMask™ Deep Red Plasma Membrane Stain (Thermo Fisher Scientific) for 10 min at 41 °C. The BM-DCs were washed 3 times in PBS and subsequently fixed for 20 min in pre-warmed 3.75% paraformaldehyde in PBS. The BM-DCs were washed 3 times in PBS and mounted on glass slides using ProLong™ Diamond Antifade Mountant with DAPI (Thermo Fisher Scientific). The analysis of the BM-DCs by confocal microscopy was performed using a Leica TCS SP5 confocal microscope and the image analysis was performed using ImageJ.

### 2.7. RT-qPCR

The BM-DCs were lysed in culture plates and RNA-purified using the RNeasy Minikit (Qiagen, Hilden, Germany) according to the manufacturer’s instructions. On-column DNase DNA digestion (Qiagen, cat. no. 79256) was performed to remove any residual contamination by genomic DNA. The purified RNA was quantified and 260/280 ratio quality checked using a Nanodrop^®^ ND1000 Spectrophotometer and subsequently reverse transcribed using the High Capacity cDNA Reverse Transcription Kit (Applied Biosystems, Waltham, MA, USA) and stored at −20 °C until further use. RT-qPCR analysis was performed using the SsoAdvanced™ Universal SYBR^®^ Green Supermix (Bio-Rad, Hercules, CA, USA) with forward and reverse primers presented in Supplementary Table S1. The relief of the PCR inhibitors was by a 25-fold dilution of cDNA. The individual qPCR reactions contained 8 µL of reaction mix, 0.25 µM of each primer and 2 µL diluted cDNA. The reactions were run as duplicates with no-RT and no-template controls. The PCR efficiencies of each run were determined using triplicates of 10-fold serial dilutions of cDNA from BM-DCs. The PCR cycles employed in the ViiA™ 7 system were 50 °C for 2 min, 95 °C for 10 min with optics off; 40 cycles of 95 °C for 15 s followed by 60 °C for 20 s with optics on. Subsequently, a melting curve stage was run for 15 s at 95 °C, 60 °C for 1 min and 95 °C for 15 sec. The Cq values obtained from the unstimulated control (media) and the treated BM-DCs were in the range of 22–37 and 16–38, respectively. The expression stability of reference genes *HMBS*, *TBP*, *HPRT1*, *GAPDH* and *RLP13* were determined based on expression (*n* = 3/treatment) and *HMBS* was ranked most stable using NormFinder [39]. All the Cq values were normalised to *HMBS* and the relative expression ratios were calculated as described [40] using the Cq values from the media-treated BM-DCs as calibrators. The results were log transformed and presented relative to the level of expression observed in media-treated BM-DCs.

### 2.8. Recombinant IBV N Protein

The construction of the pQE30 vector encoding rN, the transformation of *Eschericia coli* JM109 and the expression of rN were all done as previously described [41]. The rN protein was purified using the HisPur™ Ni-NTA Purification Kit according to the manufacturer’s instructions (Thermo Scientific™, Rockford, USA). The protein concentration of the eluted fractions was determined using the BCA™ protein assay kit (Thermo Scientific™) and the eluted fractions were analysed by 4–12% SDS-PAGE. The protein was visualised using colloid staining.

### 2.9. BM-DC Antigen Pulsing and Ex Vivo Antigen Stimulation of PBMCs

Conventional antigen-pulsing of BM-DCs was performed by incubating the BM-DCs overnight (~18 h) with RPMI-1640 (media), rN (20 µg/mL), pustulan (5 µg/mL) or rN (20 µg/mL) + pustulan (5 µg/mL). The BM-DCs were washed 3 times in ice-cold PBS and detached as described above, resuspended in RPMI-1640 supplemented with 0.1% penicillin and streptomycin (Pen–Strep) and seeded in 96-well flat-bottom plates at a concentration of 5 × 10^4^ cells/100 µL. PBMC isolation was done as previously described [42]. In brief, heparinised blood was taken from IBV-immunised MHC-matched chickens (MHC-B21, *n* = 6) and the PBMCs were isolated and resuspended at a concentration of 1 × 10^7^ cells/mL in RPMI-1640 supplemented with 10% FBS and 0.1% Pen–Strep. The PBMCs were subsequently mixed with pulsed BM-DCs in 96-well flat-bottom plates at a volume of 100 μL/well (1 × 10^6^ cells/well) yielding a BM-DC:PBMC ratio of 1:20, suggested as optimal by Carrio et al. [43]. The PBMCs were cultured alone (without BM-DCs) with RMPI-1640 (media), UV-inactivated IBV (0.15 dose/well), rN (20 µg/mL), pustulan (5 µg/mL), UV-inactivated IBV (0.15 dose/well) + pustulan (5 µg/mL), or rN (20 µg/mL) + pustulan (5 µg/mL). The cells were cultured or co-cultured for 72 h where after lymphocytes were detached with 2 mM EDTA. Lymphocytes were pelleted by centrifugation (600× *g*, 5 min) and stained in 96-well U-bottom plates as described above prior to the flow cytometry acquisition.

### 2.10. Statistical Analysis

A multiple comparison two-tailed Student’s *t*-test with Bonferroni correction was performed on the mean fluorescence intensity (MFI) flow cytometry data and the log-normally distributed qPCR and blast percentage data to estimate the differences explained by the treatments studied. For all the statistical analyses, a nominal *p*-value ≤ 0.05 was considered statistically significant. All the statistical analyses were performed in R and all the results were visualised using the *ggpubr* package for R [44].

## 3. Results

### 3.1. BM-DC MHCII Surface Expression Is Up Regulated by Pustulan, Mannan, and Chitosan

**Figure 1:**
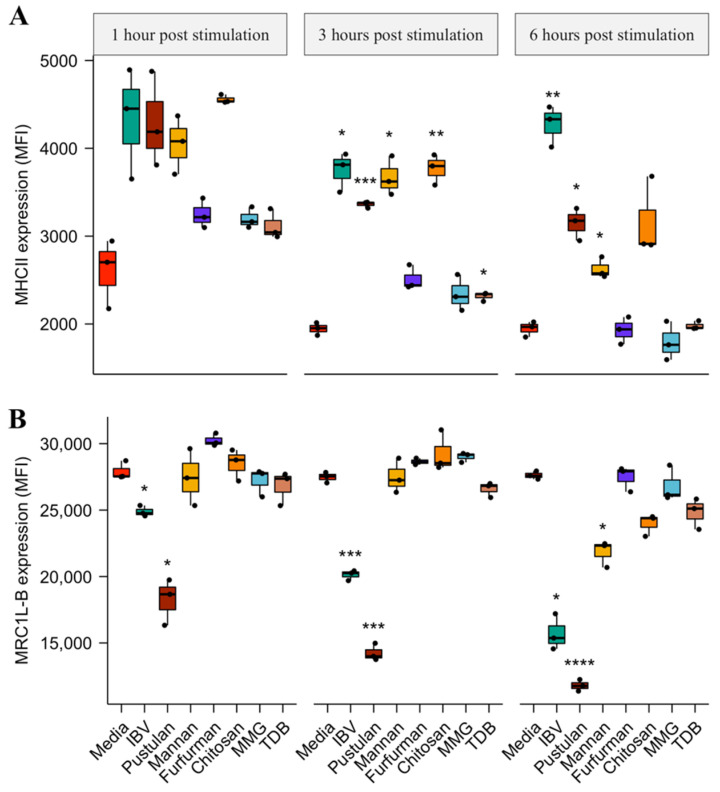
Glycan-based ligand induced effect on bone marrow-derived dendritic cell (BM-DC) MHC class II (MHCII) and MRC1L-B surface expression. BM-DC MHC-II expression (**A**) and MRC1L-B expression (**B**) are shown as the mean fluorescence intensity (MFI) after 1, 3 and 6 h post stimulation with media, UV-inactivated infectious bronchitis virus (IBV), pustulan, mannan, furfurman, chitosan, Trehalose-6,6-dibehenate (TDB) and monomycolyl glycerol (MMG). Results are presented as boxplots with individual values of biological replicates (*n* = 3) plotted. A two-tailed Student’s *t*-test with Bonferroni multiple comparison correction was employed using the media as the reference group. A *p* value < 0.05 was considered statistically significant and are indicated by asterisks. Significance level between the media and treatments are * *p* < 0.05, ** *p* < 0.01, *** *p* < 0.001, **** *p* ≤ 0.0001.

The activation of chicken BM-DCs was analysed to assess the immunostimulatory potential of the glycan-based ligands pustulan, mannan, furfurman, chitosan, MMG and TDB. These glycan-based ligands were chosen as they are commercially available and known to induce CLR-mediated APC activation in mammals. Whole UV-inactivated IBV was included as a positive control and unstimulated BM-DCs as a negative control (media) for BM-DC activation. Flow cytometry was used to assess the activation of the BM-DCs (gating strategy Supplementary Materials Figure S1). The activation of the BM-DCs was assessed by the surface expression of MHC class II (MHCII), MRC1L-B, and CD45 (Supplementary Materials Figure S2) after 1, 3 and 6 h post stimulation as the surface expression kinetics of MHCII in response to the glycan-based ligands were not known. The MHCII surface expression 1 h post stimulation did not differ significantly for any of the treatments of BM-DCs compared to the media-treated BM-DCs (Figure 1A). The MHCII surface expression 3 h post stimulation was, compared to the media-treated BM-DCs, significantly different for the UV-inactivated IBV (1.9 × increase, *p* = 0.05), pustulan (1.7 × increase, *p* = 0.001), mannan (1.8 × increase, *p* = 0.05), chitosan (1.9 × increase, *p* = 0.01) and TDB-treated BM-DCs (1.19 × increase, *p* = 0.05). Similarly, the MHCII expression 6 h post stimulation was, compared to the media-treated BM-DCs, significantly different for UV-inactivated IBV (2.2 × increase, *p* = 0.01), pustulan (1.6 × increase, *p* = 0.05) and mannan-treated BM-DCs (1.3 × increase, *p* = 0.03).

The cell surface expression of MRC1L-B changed over time, compared to the media-treated BM-DCs, for the pustulan-treated BM-DCs after 1 h (1.5 × decrease, *p* = 0.03), 3 h (1.9 × decrease, *p* = 0.0003) and 6 h (2.3 × decrease, *p* = 0.00002) post stimulation and for UV-inactivated IBV-treated BM-DCs after 1 h (1.1 × decrease, *p* = 0.04), 3 h (1.4 × decrease, *p* = 0.0001) and 6 h (1.8 × decrease, *p* = 0.02) post stimulation (Figure 1B). Furthermore, the cell surface expression of MRC1L-B, compared to the media-treated BM-DCs, was significantly different for the mannan-treated BM-DCs 6 h post stimulation (1.3 × decrease, *p* = 0.04). CD45 surface expression showed only minor variations induced by the glycan-based ligands over time. However, the CD45 surface expression 6 h post stimulation, compared to the media-treated BM-DCs, was significantly different for the pustulan-treated BM-DCs (1.3 × decrease, *p* = 0.02) (Supplementary Materials Figure S2).

### 3.2. Pustulan and Mannan Induce a Gene Expression of Proinflammatory Cytokines

**Figure 2:**
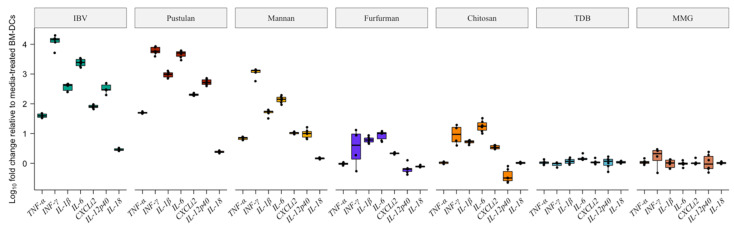
BM-DC expression of the proinflammatory cytokines at 3 h post stimulation. RNA was purified from BM-DCs 3 h post stimulation with UV-inactivated IBV (IBV), pustulan, mannan, furfurman, chitosan, TDB and MMG. Expression of cytokines was determined by RT-qPCR. Cq values were determined from the technical replicates (*n* = 2) for each biological replicate (*n* = 3). Results are shown as boxplots in a logarithmic scale as a fold change relative to the level of expression observed in the media-treated BM-DCs with the individual values of the biological replicates (*n* = 3) plotted.

The highest increase in the MHCII surface expression, compared to the media-treated BM-DCs, was observed 3 h post stimulation of the BM-DCs treated with UV-inactivated IBV, pustulan, mannan and chitosan. Therefore, the mRNA expression of the proinflammatory cytokines was analysed by RT-qPCR at 3 h post stimulation. The expression of the proinflammatory cytokines induced by the UV-inactivated IBV, pustulan, mannan, furfurman, chitosan, MMG and TDB, is shown as a fold change relative to the expression of proinflammatory cytokines induced by the media (Figure 2). The BM-DCs treated with UV-inactivated IBV, pustulan and mannan showed an up regulation of *TNF-α*, *IFN-γ*, *IL-1β*, *IL-6*, *CXCLi2*, *IL-12p40*, and *IL-18*. The BM-DCs treated with furfurman showed an up regulation of *IL-1β*, *IL-6*, and *CXCLi2*, while the BM-DCs treated with chitosan showed an up regulation of *IFN-γ*, *IL-1β*, *IL-6*, *IL-8*. MMG and TDB induced only minor differences in the expression for any of the analysed cytokines compared to the media at 3 h post stimulation.

### 3.3. Cation-Dependent Increase in Endocytosis of FITC-Labelled BSA by Pustulan and Chitosan

**Figure 3:**
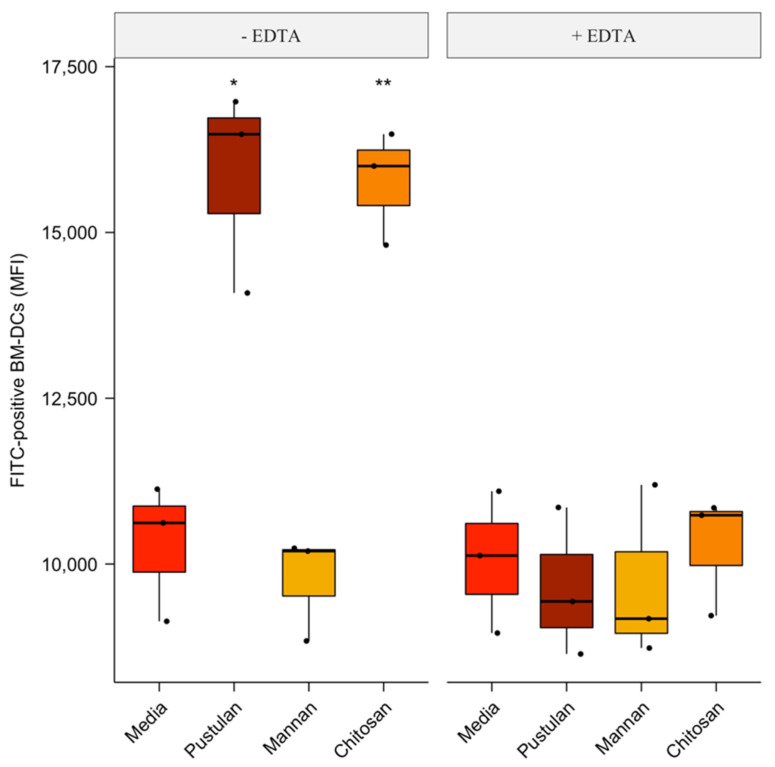
Cation-dependent effect of the glycan-based ligands on the endocytosis of FITC-labelled BSA by BM-DCs. Uptake of FITC-labelled BSA (10 µg/mL) in the presence of pustulan (100 µg/mL) or mannan (500 µg/mL) by BM-DCs preincubated for 15 min with or without EDTA (10 mM) was analysed by flow cytometry after 60 min Results are presented as boxplots showing the mean fluorescence intensity of the FITC-positive BM-DCs with individual values of biological replicates (*n* = 3) plotted. A two-tailed Student’s *t*-test with Bonferroni multiple comparison correction was employed using the media as a reference group. A *p* value ≤ 0.05 was considered statistically significant and are indicated by asterisks. Significance level between the media and treatments is * *p* ≤ 0.05, ** *p* ≤ 0.01.

The effect of pustulan, mannan and chitosan on endocytosis by BM-DCs was analysed as these glycan-based ligands appeared to be BM-DC activators. The BM-DCs were incubated with FITC-BSA mixed with either pustulan, mannan, chitosan or the media. The cation chelator, EDTA, was included to study the effect of cation depletion on endocytosis. The endocytosis of FITC-BSA was analysed by flow cytometry and the intracellular location of FITC-BSA verified by confocal microscopy. Endocytosis was measured as an increase in MFI above the 4 °C negative control (not shown). Endocytosis, compared to the media-treated BM-DCs, was significantly different for the pustulan- (1.5 × increase, *p* = 0.028) and the chitosan-treated BM-DCs (1.5 × increase, *p* = 0.007) (Figure 3). Furthermore, the cation depletion by the addition of EDTA abrogated the pustulan and chitosan-induced increase in FITC MFI. No difference in endocytic potential was observed between the mannan and the media-treated BM-DCs. The intracellular localisation of FITC-BSA was verified by confocal microscopy (Supplementary Figure S3).

### 3.4. Pustulan Enhances Activation of Recall Antigen-Specific CD4^+^ But Not CD8^+^ T Cells in PBMC Cultures

**Figure 4:**
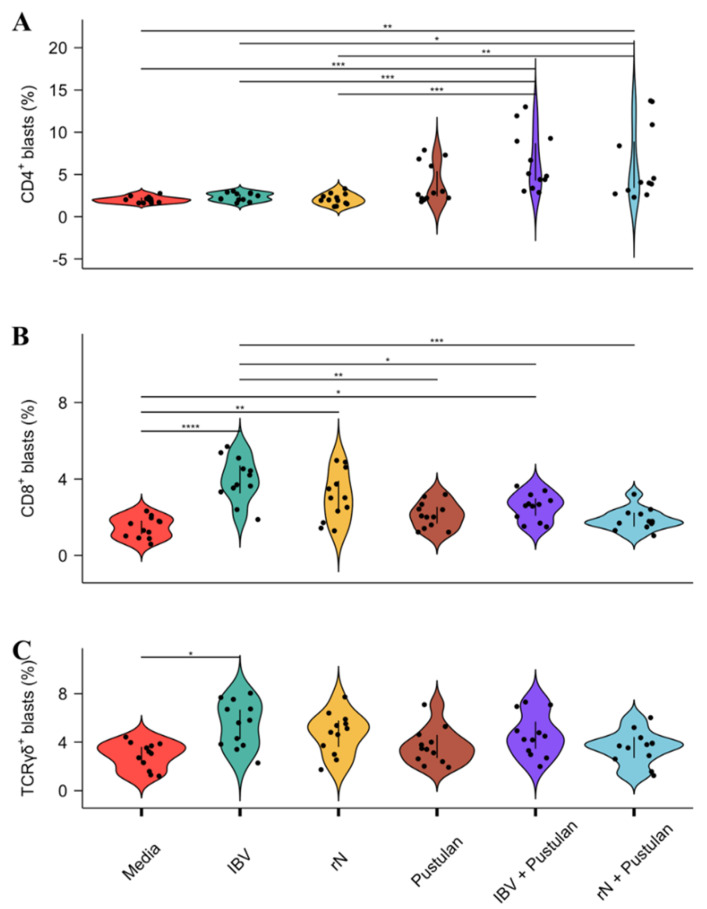
Blast transformation of the CD4^+^ (**A**), CD8^+^ (**B**), and TCRγδ^+^ (**C**) T cells induced by the pustulan-adjuvanted antigen. PBMCs isolated from the heparin-stabilised blood of IBV-vaccinated MHC-matched chickens (*n* = 6) were seeded in flat-bottom 96-well plates (1 × 10^6^ cells/well) and treated for 72 h with RPMI-1640 (media), UV-inactivated IBV (0.15 dose/well), rN (20 µg/mL), pustulan (5 µg/mL), UV-inactivated IBV (0.15 dose/well) + pustulan (5 µg/mL), or rN (20 µg/mL) + pustulan (5 µg/mL). Blast transformations of the CD4^+^, CD8^+^, and TCRγδ^+^ T cells were analysed by flow cytometry. Results are presented as violin plots with 95% confidence intervals showing the individual blast % values plotted of the technical replicates (*n* = 2/treatment) from the biological replicates (n = 6/treatment). The data were log-transformed and a two-tailed Student’s *t*-test with Bonferroni multiple comparison correction was employed. A *p* value ≤ 0.05 was considered statically significant and differences between the treatments are indicated by bars. Significance level is indicated by asterisks * *p* ≤ 0.05, ** *p* ≤ 0.01, *** *p* ≤ 0.001, **** *p* ≤ 0.0001.

As pustulan increased the MHCII surface expression, the expression of proinflammatory cytokines and endocytosis, a potential immunopotentiating effect of pustulan on the recall activation of PBMCs from IBV-immunised chickens, was first analysed without BM-DCs, thereby relying on APCs in the PBMC samples such as monocytes and/or B cells. The verification of purified rN was performed by 4%–12% SDS-PAGE (Supplementary Figure S4). The concentration of pustulan for the *ex vivo* blast transformation experiments was determined by titration on BM-DCs by assessing the MHCII cell surface expression and the percentage of viable cells after an overnight incubation (Supplementary Figure S5). As 10 µg/mL pustulan increased the surface expression of MHCII approximately two-fold, but reduced the percentage of viable MHCII-positive cells by 20%, we used 5 µg/mL pustulan to increase the number of viable cells after overnight incubation. The blast transformation of CD4^+^, CD8^+^ and TCRγδ^+^ T cells was analysed by flow cytometry and shown as a blast’s percentage (gating strategy shown in Supplementary Figure S6). Pustulan-adjuvanted rN induced a significantly different CD4^+^ blast percentage compared to the media (3× increase, *p* = 0.005), the UV-inactivated IBV (2.6× increase, *p* = 0.02) and the rN (3× increase, *p* = 0.005) (Figure 4A). Moreover, the pustulan-adjuvanted UV-inactivated IBV induced a significantly different CD4^+^ blast percentage compared to the media (3.2× increase, *p* = 0.0002), the UV-inactivated IBV (2.8× increase, *p* = 0.0007) and the rN (3.1× increase, *p* = 0.0002).

UV-inactivated IBV induced a significantly different CD8^+^ blast percentage compared to the media (2.7× increase, *p* = 0.00002) (Figure 4B). Similarly, rN induced a significantly different CD8^+^ blast percentage compared to the media (2.1× increase, *p* = 0. 008).

Moreover, UV-inactivated IBV induced a significantly different CD8^+^ blast percentage compared to pustulan (1.9× increase, *p* = 0.001), pustulan-adjuvanted UV-inactivated IBV (1.6× increase, *p* = 0.02), and the pustulan-adjuvanted rN (2.1× increase *p* = 0.00009). Only the UV-inactivated IBV induced a significantly different TCRγδ+ blast percentage compared to the media (1.9× increase, *p* = 0.015) (Figure 4C).

### 3.5. Pustulan Augments BM-DC-Induced Activation of Recall Antigen-Specific CD4^+^ T Cells

**Figure 5:**
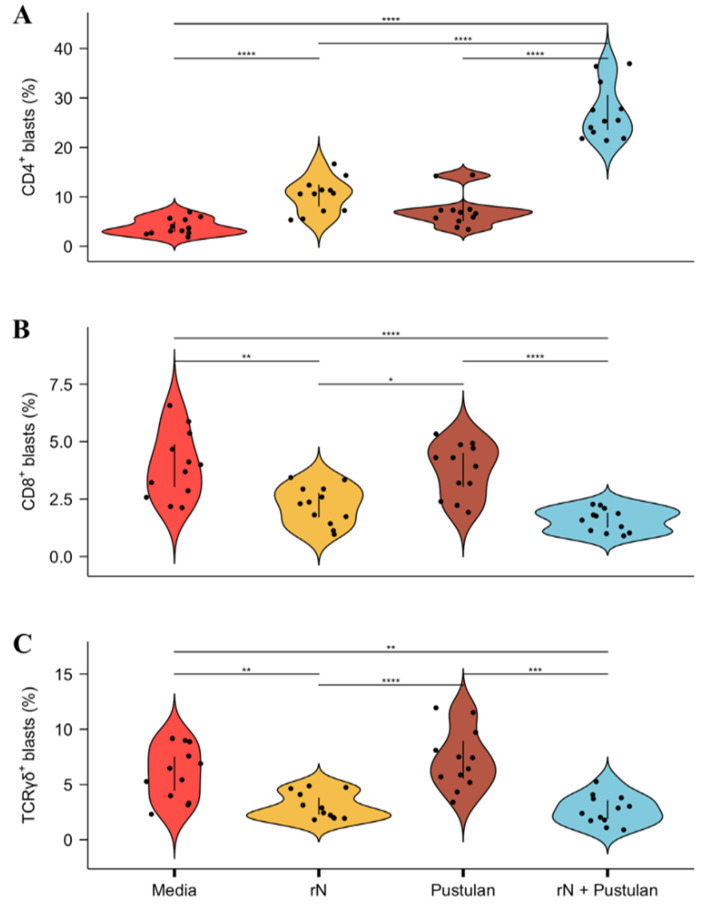
Blast transformation of the CD4^+^ (**A**), CD8^+^ (**B**) and the TCRγδ^+^ (**C**) T cells induced by the antigen-pulsed BM-DCs. BM-DCs were pulsed overnight with the media, recombinant IBV N protein (rN) (20 µg/mL), pustulan (5 µg/mL), or rN (20 µg/mL) + pustulan (5 µg/mL). Pulsed BM-DCs were washed and co-cultured for 72 h in flat-bottom 96-well plates with PBMCs isolated from the heparin-stabilised blood of IBV-vaccinated MHC-matched chickens (*n* = 6). The blast transformations of the CD4^+^, CD8^+^ and TCRγδ^+^ T cells were analysed by flow cytometry. Results are presented as violin plots with 95% confidence intervals showing the individual blast % values plotted of the technical replicates (*n* = 2/treatment) from the biological replicates (*n* = 6/treatment). The data were log-transformed and a two-tailed Student’s *t*-test with Bonferroni multiple comparison correction was employed. A *p* value ≤ 0.05 was considered statically significant and differences between the treatments are indicated by bars. Significance level is indicated by asterisks * *p* ≤ 0.05, ** *p* ≤ 0.01, *** *p* ≤ 0.001, **** *p* ≤ 0.0001.

Recombinant IBV N protein may simulate a subunit vaccine. Thus, the potential immunopotentiating effect of pustulan on the recall activation of PBMCs from IBV-immunised MHC-matched chickens was, furthermore, analysed using rN-pulsed BM-DCs. The blast transformations of the CD4^+^, CD8^+^, and TCRγδ^+^ T cells induced by the antigen-pulsed BM-DCs were analysed by flow cytometry and expressed as blast transformation frequencies (gating strategy shown in Supplementary Figure S6). A significantly different CD4^+^ blast percentage was, compared to the media-pulsed BM-DCs, induced by rN (2.6× increase, *p* < 0.00001) and pustulan-adjuvanted rN-pulsed BM-DCs (6.8× increase, *p* < 0.00001) (Figure 5A). Moreover, the pustulan-adjuvanted rN-pulsed BM-DCs induced a significantly different CD4^+^ blast percentage compared to the pustulan- (3.7× increase, *p* < 0.00001) and the rN-pulsed BM-DCs (2.6× increase, *p* < 0.00001).

A significantly different CD8^+^ blast percentage was induced by the rN-pulsed BM-DCs compared to the media (1.8× decrease, *p* = 0.006) and the pustulan-pulsed BM-DCs (1.7× decrease, *p* = 0.006) (Figure 5B). Likewise, the pustulan-adjuvanted rN-pulsed BM-DCs induced a significantly different CD8^+^ blast percentage compared to the media (2.5× decrease, *p* = 0.00002) and pustulan-pulsed BM-DCs (2.4× decrease, *p* = 0.00002). The same effect was observed for the TCRγδ^+^ blast percentage (Figure 5C). A significantly different TCRγδ^+^ blast percentage was induced by the rN-pulsed BM-DCs compared to the media (2× decrease, *p* = 0.002) and the pustulan-pulsed BM-DCs (2.4× decrease, *p* = 0.00007). Similarly, the pustulan-adjuvanted rN-pulsed BM-DCs induced a significantly different TCRγδ^+^ blast percentage compared to the media (2.2× decrease, *p* = 0.002) and the pustulan-pulsed BM-DCs (2.7× decrease, *p* = 0.0001).

## 4. Discussion

The aim of this study was to apply an in vitro model using chicken BM-DCs to screen CLR agonists for immunostimulatory potential in order to identify potential glycan-based adjuvants for chicken vaccination. The β-1-6-(D)-glucan, pustulan, activated chicken BM-DCs, increased the endocytic capacity of BM-DCs and enhanced the recall activation of IBV-specific CD4^+^ T cells ex vivo. This study is the first to describe a potential immunopotentiating effect of pustulan in vitro using chicken BM-DCs, indicating the potential of pustulan as a glycan-based adjuvant, which may boost inactivated or subunit chicken vaccines in vivo.

Chicken BM-DCs generated using chicken serum supplementation have previously been characterised in a study by van den Biggelaar et al. as a heterogenous population in respect to the maturation state [45]. The study, moreover, showed that the use of 10% FBS instead of chicken serum supplementation resulted in a more homogenous population. Thus, the results presented in this study did not originate from a heterogenous population with distinct cell types as observed for the murine BM-DC cultures [46]. The overall phenotype of the BM-DCs derived using the approach employed in this study are therefore comparable to previous reports [47,48,49]. Mannan- and pustulan- treated BM-DCs increased MHCII surface expression (Figure 1A) and the expression of proinflammatory cytokines (Figure 2). The LPS-induced maturation of chicken BM-DC led to the increased surface expression of MHCII and the expression of proinflammatory cytokines such as *TNF*-α, INF-*γ*, *IL-6*, *IL-1β, CXCLi2*, and *IL-12p40* [50,51]. This was similar to the effects of the LPS-induced maturation of mammalian BM-DCs [52]. Thus, our results strongly indicate that both mannan and pustulan exert an immunostimulatory effect on chicken BM-DCs, which may lead to DC maturation. Chitosan increased MHCII surface expression (Figure 1A) but did not induce a high expression of proinflammatory cytokines in comparison to mannan and pustulan (Figure 2). We focused on the qualitative screening of glycans and thus, did not titrate the concentration of chitosan to find an optimal concentration for stimulation. Thus, we cannot exclude that chitosan at higher concentrations might induce expression levels of proinflammatory cytokines similar to mannan and pustulan.

Pustulan is a 20 kDa linear β-1-6-(D)-glucan derived from the cell wall of lichen *L. pustulata*. Studies on the immunostimulatory potential of β-glucans in mammals are mainly focused on β-1-3-(D)-glucans such as curdlan and zymosan [53]. To date, findings regarding the immunostimulatory effect of pustulan are limited and contradictory. A study on cytokine production in human whole blood showed a stronger cytokine response induced by β-1-6-(D)-glucans than by β-1-3-(D)-glucans [33]. On the other hand, a study of lichen-derived polysaccharide maturation of human BM-DCs reported pustulan to induce a high secretion of IL-10 and a low secretion of IL12-p40, suggesting an anti-inflammatory effect of pustulan. Thus, the apparent immunostimulatory effect of pustulan in chicken might indicate that chicken BM-DCs express PRRs, with affinity for pustulan, not present in mammals. This is in agreement with our earlier finding that chickens and mammals only share few CLR orthologues and that the chicken CLR gene repertoire differs significantly from the mammalian repertoire [54].

Pustulan increased the capacity of chicken BM-DCs to endocytose BSA in a cation-dependent manner (Figure 3). Interestingly, in contrast to murine studies, no effect of mannan on endocytosis was observed. In mice, the addition of mannan enhances the endocytosis of fluorescent labelled bacteria by BM-DCs [55] and fluorescent microbeads by macrophages [56]. In humans and mice, pustulan is recognised by Dectin-1 [57], which is a receptor for β-glucans present in the cell wall of several fungal pathogens including *Aspergillus*, *Candida*, *Coccidioides* and *Pneumocystis* [58]. The engagement of Dectin-1 induced signalling through an intracellularly located single-copy immunoreceptor tyrosine-based activation motif (hemITAM) [59] which led to altered cellular responses such as enhanced phagocytosis, DC maturation, antigen presentation and the expression of cytokines [60,61]. Moreover, TLR 2/4 and complement receptor 3 (CR3/CD11b) have also been shown to bind β-glucans and promote phagocytosis and cytokine expression in mammalian APCs [62,63]. However, Dectin-1, TLR2/4 and CR3 bind β-glucans in a cation-independent manner [64,65,66]. The pustulan-induced increase in endocytosis of BSA was abrogated by EDTA. Moreover, chicken Dectin-1 or CR3 orthologues have not been identified to date [54,67]. In chicken, pustulan might thus be recognised by a PRR not present in mammals such as the recently reported Group II chicken CLRs CLEC17AL-A and -B [54].

The effect of pustulan on IBV-antigen-specific T cell recall responses was assessed using PBMCs from IBV-immunised chickens either treated with pustulan-adjuvanted UV-inactivated IBV or rN, (Figure 4) or co-cultured with MHC-matched BM-DCs pulsed with pustulan-adjuvanted rN (Figure 5). Additional bands shown on the SDS-PAGE (Supplementary Figure S4) belong to a laboratory *Escherichia coli* strain, which does not exist in the field [41]. Although antigens may still be shared between laboratory and field strains, we may assume that the significant response was due to memory T cells being activated by rN. The recall proliferation of PBMC from vaccinated chickens has previously been shown to be higher compared to naive chickens 21 days post booster vaccination [68]. The level of antibody-secreting-cells in PBMC samples has been shown to be approximately 0.5 antibody-secreting-cells/10^6^ cells 2.5 months post IBV-infection [69]. Here, the PBMCs were sampled from IBV-immunised chickens eight months post booster vaccination. Thus, although recall proliferation is higher for PBMC from immunised chickens compared to naïve ones, the level of IBV-specific T cells (as well as plasma cells) was expected to be relatively low and the T cell response observed cannot be ruled out as being generated by the activation of naive T cells.

Although humoral immunity is important in IBV vaccine-induced protection, there is a general lack of correlation between protection and serum anti-IBV titres [2,3], hence, our focus was on the cell-mediated immunity and activation of T cells. Pustulan-adjuvanted UV-inactivated IBV or rN induced a significant blast transformation of CD4^+^ (Figure 4). Moreover, PBMCs co-cultured with BM-DCs pulsed with pustulan-adjuvanted rN showed an augmented blast transformation of CD4^+^ T cells compared to the PBMCs treated with pustulan-adjuvanted rN (Figure 5). This indicates that pustulan not only potentiates the recall activation of IBV-antigen-specific CD4^+^ T cells in PBMC cultures, but also augments antigen-presentation to IBV-antigen-specific CD4^+^ T cells by chicken BM-DCs. Whether the increase in the endocytic capacity of chicken BM-DCs (Figure 3) has biological relevance in vivo remains to be investigated. However, the pustulan-induced activation stimuli (Figure 2) and increased endocytic capacity of chicken BM-DCs (Figure 3) might explain the increased efficacy of the observed antigen-presentation to IBV-specific CD4^+^ T cells.

PBMCs treated with pustulan-adjuvanted UV-inactivated IBV or rN did not induce CD8^+^ blast transformation above the background level (Figure 4). Likewise, BM-DCs pulsed with pustulan-adjuvanted rN did not induce CD8^+^ blast transformation above the background level (Figure 5). However, PBMCs treated with antigen alone induced CD8^+^ blast transformation above the background level (Figure 4), indicating a potential pustulan-induced abrogation of IBV-antigen-specific CD8^+^ T cell recall activation. Hence, pustulan might enhance the efficacy of MHCII antigen presentation, while lowering the efficacy of MHCI antigen presentation (cross-presentation). The expression of MHCI was not investigated in this study to address cross-presentation, as CD8^+^ blast transformation is a better proxy for MHCI antigen presentation. In mammals, the efficacy of cross-presentation depends largely on the type of DC subset [70]. In addition, the preference for antigen size by PRRs expressed by the DC subsets has also been shown to influence the cross-presentation of antigens. In a study using human Langerhans cells (LCs), a CLR, langerin, was shown to facilitate the phagocytosis of small glycan-modified synthetic peptide antigens and efficiently activate CD4^+^ T cells and CD8^+^ cells [71]. In contrast, large glycan-modified antigen-containing liposomes were not phagocytosed through the engagement of Langerin. However, targeting the antigen-containing liposomes to LCs using anti-langerin increased phagocytosis and CD4^+^ T cell activation, but not CD8^+^ T cell activation. Therefore, in humans, antigen properties such as size are important for successful DC subset targeting and moreover, subsequent cross-presentation. Hence, from this study we cannot rule out that β-glucans of other molecular weights than pustulan or that the engagement of other chicken DC subtypes in vivo might result in a more efficient cross-presentation.

## 5. Conclusions

In conclusion, we successfully applied an in vitro model using chicken BM-DCs to screen glycans for potential immunopotentiating effects. We found an immunopotentiating effect in vitro of the β-1-6-glucan, pustulan, as measured by the increased surface expression of MHCII, a strong proinflammatory cytokine response, increased endocytic capacity and an increased recall activation of CD4^+^ T cells. Future in vivo studies are, however, required to determine the benefits of pustulan as a glycan-based adjuvant. Nevertheless, this study indicates that glycans may be used for targeting antigens to chicken DCs in vivo through surface-expressed CLRs. Thus, pustulan as well as other β-glucans hold promise as glycan-based adjuvants, which may potentially boost the in vivo efficacy of subunit or inactivated viral vaccines to protect against viral pathogens in chickens such as IBV and thereby limit the risk of reversion to virulence of live-attenuated viral vaccines.

## Supplementary Materials

The following are available online at https://www.mdpi.com/2076-393X/8/2/226/s1, Figure S1: Gating strategies for flow cytometric analysis of glycan-based ligand stimulation. The gating strategy used for Figure 1 was: FSC/SSC defined bone marrow-derived dendritic cells (P1), FSC-H/FSH-A single cells (Singlets), dead cell exclusion using Near-IR live/dead cells stain (Viable cells), CD45+ positive cells, CD45+KUL01+MHCII+ cells, Figure S2: Glycan-based ligand induced effect on BM-DC CD45 surface expression. BM-DC CD45 surface expression is shown as mean fluorescence intensity (MFI) 1, 3, and 6 h post stimulation with media, UV-inactivated IBV (IBV), pustulan, mannan, furfurman, chitosan, TDB, and MMG. Results are presented as boxplots with individual values of biological replicates (*n* = 3) plotted. A two-tailed Student’s t-test with Bonferroni multiple comparison correction was employed using media as reference group. A *p* value ≤ 0.05 was considered statically significant and are indicated by asterisks. Significance level between media and treatments are * *p* ≤ 0.05, ** *p* ≤ 0.01, *** *p* ≤ 0.001, **** *p* ≤ 0.0001, Figure S3: Confocal microscopy and histograms of endocytosed FITC-labelled BSA (FITC-BSA). To verify phagocytosis, bone marrow-derived dendritic cells were seeded on collagen-coated coverslips in 12-well plates and incubated with FITC-BSA (green) supplemented with either (A) RPMI-1640, (B) pustulan (100 μg/mL), (C) mannan (500 μg/mL), or (D) chitosan (200 μg/mL). Phagocytosis was carried out for 60 min. at 41 °C. The cells were stained with fresh CellMask™ Deep Red Plasma membrane Stain (red) for 15 min at 41 °C and fixed in 3.75% paraformaldehyde. Fixed cells were mounted on glass slides using ProLong™ Diamond Antifade Mountant with DAPI (blue). Analysis by confocal microscopy was performed using a Leica TCS SP5 confocal microscope. Overlay histograms showing differences in endocytosed FITC-BSA (MFI) (E) was made using FlowJo, Figure S4: SDS-PAGE of recombinant IBV N protein (rN) eluted fractions. The rN protein was purified using the HisPur™ Ni-NTA Purification Kit according to manufacturers instructions. Protein concentration of the eluted fractions was determined using the BCATM protein assay kit and the eluted fractions with high protein concentration were analysed by 4−12% SDS-PAGE. Protein was visualised using colloid staining. The 54-kDa rN fragment was detected in the eluted fractions. The well were loaded with (1) fraction 1 + DTT, (2) fraction 2 + DTT, (3) fraction 1, and (4) fraction 2, Figure S5: Titration of pustulan to determine optimal concentration for overnight stimulation of bone marrow-derived dendritic cells. (A) MHCII cell surface expression (MFI) and (B) viable cells (MHCII positive events) was assessed after an overnight incubation with either 0 μg (blue), 10 μg, 25 μg, 50 μg, 75 μg, or 100μg pustulan (yellow). Results are shown as boxplots with individual values of biological replicates (*n* = 3) plotted, Figure S6: Gating strategies for flow cytometric analysis of ex vivo recall antigen-stimulation of peripheral blood monocytes. The gating strategy used for Figure 4 was: SSC-A/FSC-H defined lymphocytes intact cells), FSC-H/FSH-A single cells (Singlets), dead cell exclusion using LIVE/DEAD™ Fixable Near-IR Dead Cell Stain, TCR1^+^ T cells, SSC-H/FSC-H defined TCR1^+^ blasts, CD4^+^ T cells, CD4^+^ blasts, CD8α^+^ T cells, CD8α^+^ blasts, CD4^+^CD8α^+^ T cells, CD4^+^CD8α^+^ blasts. Example shows rN + pustulan treated PBMCs. Table S1: Primers used for cytokine mRNA expression analysis in chicken bone marrow-derived dendritic cells.
