# Supplementary material for: Pustulan Activates Chicken Bone Marrow-Derived Dendritic Cells In Vitro and Promotes Ex Vivo CD4+ T Cell Recall Response to Infectious Bronchitis Virus
Source: Vaccines (Basel). 2020 May 15;8(2):226. doi: 10.3390/vaccines8020226 (PMC7349971; doi:10.3390/vaccines8020226)
Supplement: Supplementary file 1 [file vaccines-08-00226-s001.zip › vaccines-748283 sup figure .pdf]

vaccines

Supplementary Material

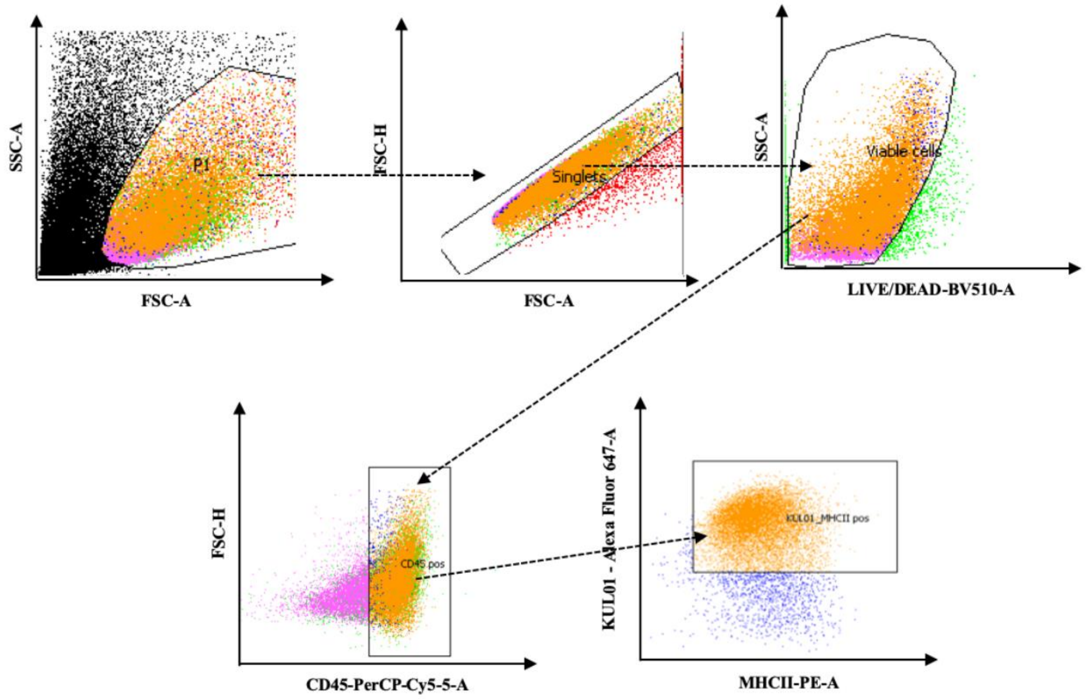

Figure S1. Gating strategies for flow cytometric analysis of glycan-based ligand stimulation. The gating strategy used for Fig. 1 was: FSC/SSC defined bone marrow-derived dendritic cells (P1), FSC-H/FSH-A single cells (Singlets), dead cell exclusion using Near-IR live/dead cells stain (Viable cells), CD45^{+} positive cells, CD45^{+}KUL01^{+}MHCII^{+} cells.

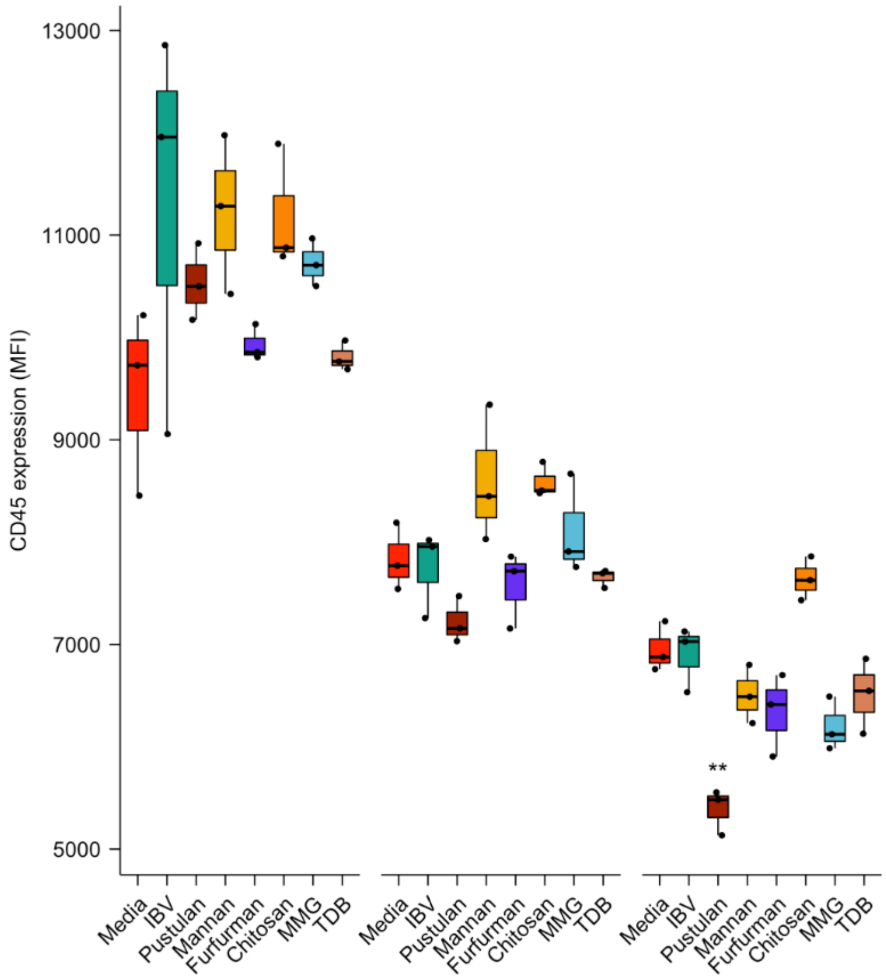

Figure S2. Glycan-based ligand induced effect on BM-DC CD45 surface expression. BM-DC CD45 surface expression is shown as mean fluorescence intensity (MFI) 1, 3, and 6 hours post stimulation with media, UV-inactivated IBV (IBV), pustulan, mannan, furfurman, chitosan, TDB, and MMG. Results are presented as boxplots with individual values of biological replicates (n = 3) plotted. A two-tailed Student's t-test with Bonferroni multiple comparison correction was employed using media as reference group. A p value ≤ 0.05 was considered statically significant and are indicated by asterisks. Significance level between media and treatments are p ≤ 0.05 *, p ≤ 0.01 **, p ≤ 0.001 ***.

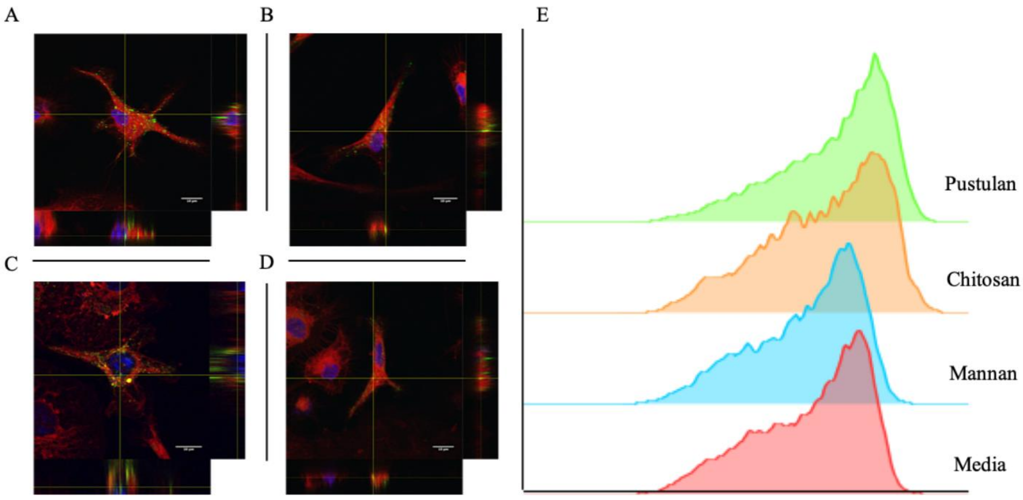

Figure S3. Confocal microscopy and histograms of endocytosed FITC-labelled BSA (FITC-BSA). To verify phagocytosis, bone marrow-derived dendritic cells were seeded on collagen-coated coverslips in 12-well plates and incubated with FITC-BSA (green) supplemented with either (**A**) RPMI-1640, (**B**) pustulan (100 µg/mL), (**C**) mannan (500 µg/mL), or (**D**) chitosan (200 µg/mL). Phagocytosis was carried out for 60 min. at 41°C. The cells were stained with fresh CellMask™ Deep Red Plasma membrane Stain (red) for 15 min at 41°C and fixed in 3.75% paraformaldehyde. Fixed cells were mounted on glass slides using ProLong™ Diamond Antifade Mountant with DAPI (blue). Analysis by confocal microscopy was performed using a Leica TCS SP5 confocal microscope. Overlay histograms showing differences in endocytosed FITC-BSA (MFI) (**E**) was made using FlowJo.

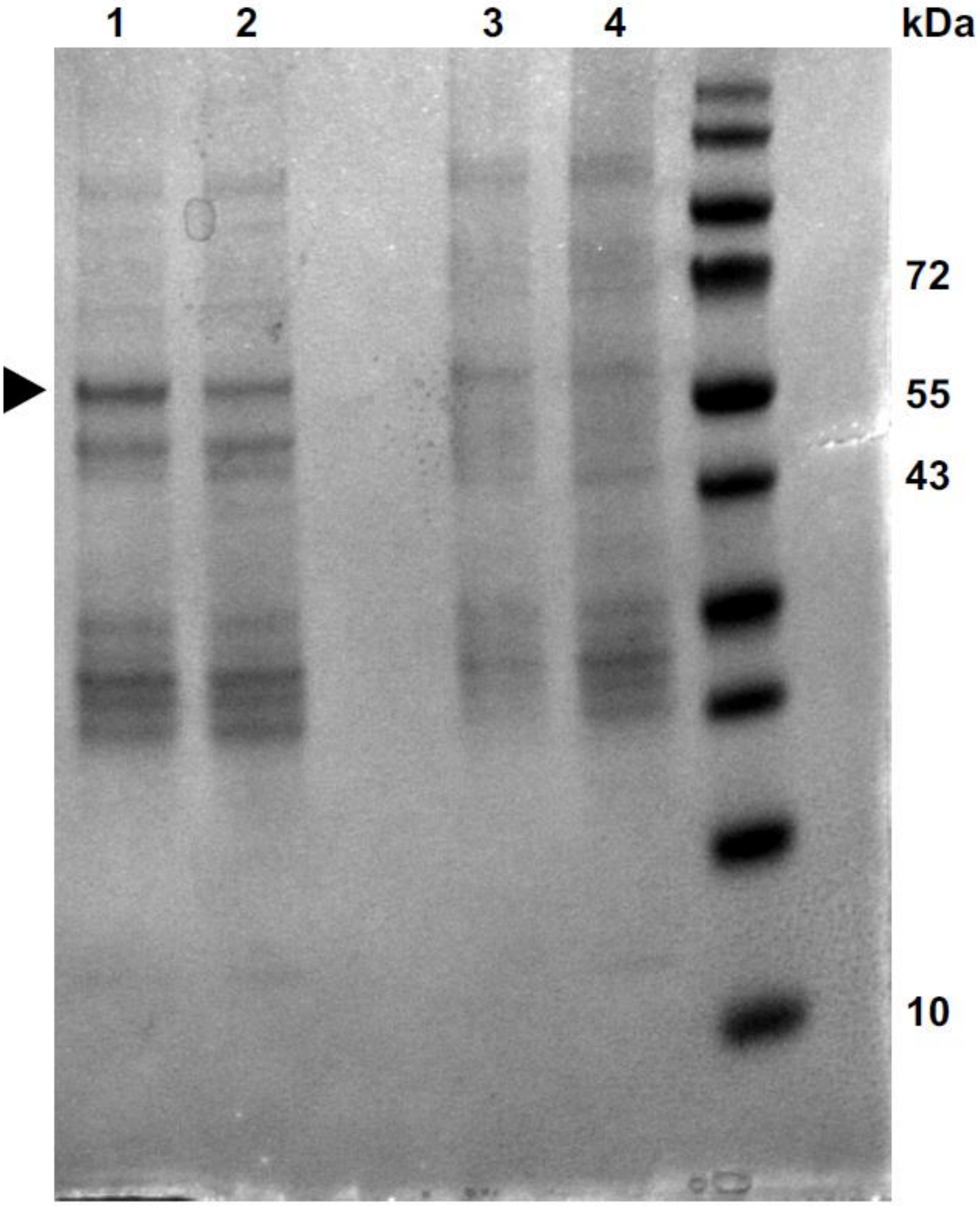

Figure S4. SDS-PAGE of recombinant IBV N protein (rN) eluted fractions. The rN protein was purified using the HisPur™ Ni-NTA Purification Kit according to manufacturers instructions. Protein concentration of the eluted fractions was determined using the BCA™ protein assay kit and the eluted fractions with high protein concentration were analysed by 4-12% SDS-PAGE. Protein was visualised using colloid staining. The 54-kDa rN fragment was detected in the eluted fractions. The well were loaded with (**1**) fraction 1 + DTT, (**2**) fraction 2 + DTT, (**3**) fraction 1, and (**4**) fraction 2. .

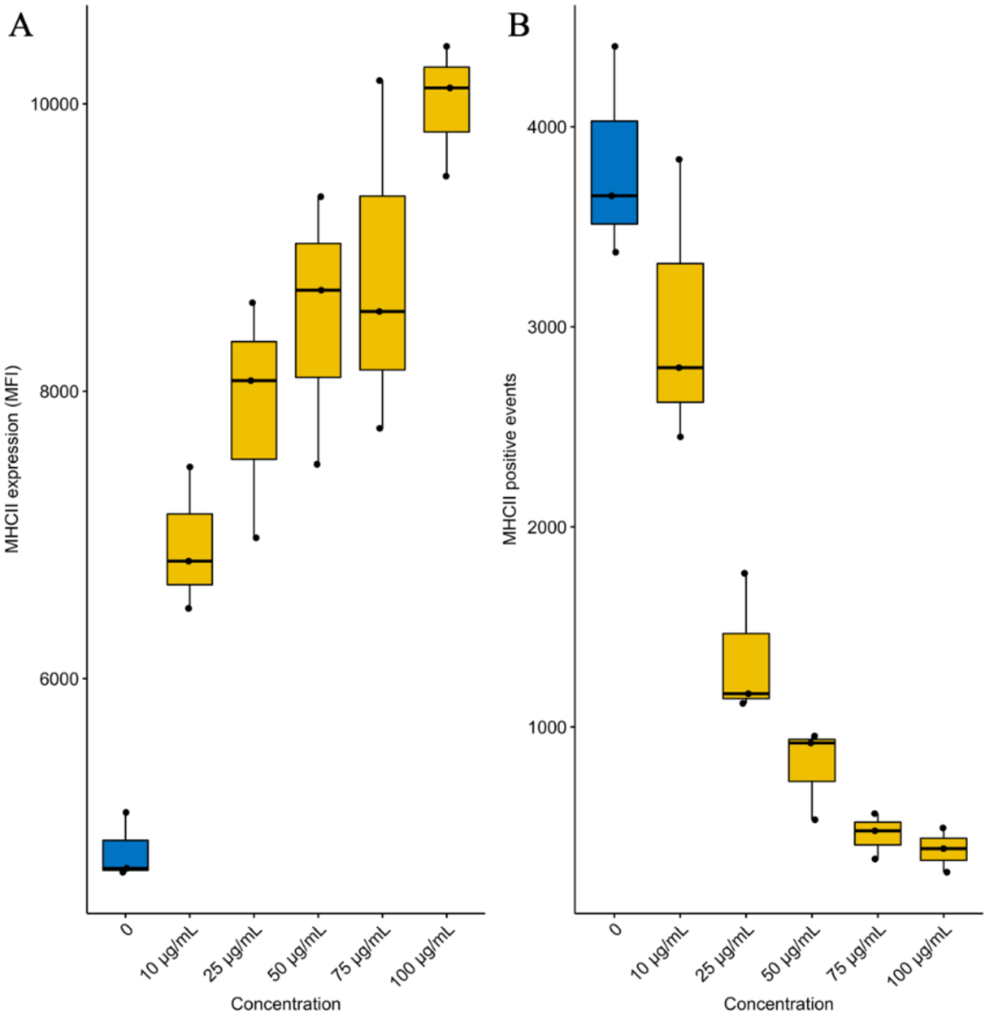

Figure S5. Titration of pustulan to determine optimal concentration for overnight stimulation of bone marrow-derived dendritic cells. (**A**) MHCII cell surface expression (MFI) and (**B**) viable cells (MHCII positive events) was assessed after an overnight incubation with either 0 µg (blue), 10 µg, 25 µg, 50 µg, 75 µg, or 100µg pustulan (yellow). Results are shown as boxplots with individual values of biological replicates (n = 3) plotted.

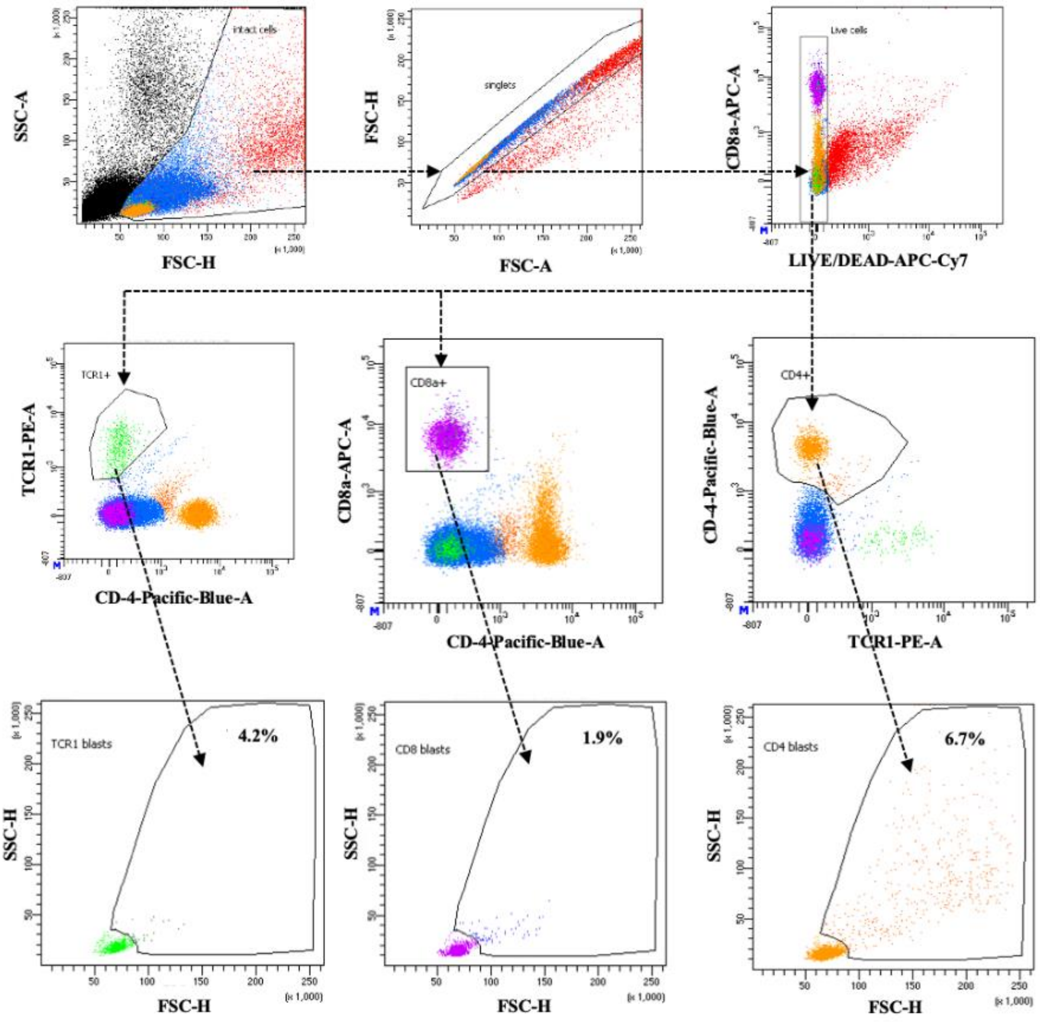

Figure S6. Gating strategies for flow cytometric analysis of *ex vivo* recall antigen-stimulation of peripheral blood monocytes. The gating strategy used for Fig. 4. was: SSC-A/FSC-H defined lymphocytes intact cells), FSC-H/FSH-A single cells (Singlets), dead cell exclusion using LIVE/DEAD™ Fixable Near-IR Dead Cell Stain, TCR1$^+$ T cells, SSC-H/FSC-H defined TCR1$^+$ blasts, CD4$^+$ T cells, CD4$^+$ blasts, CD8α$^+$ T cells, CD8α$^+$ blasts, CD4$^+$CD8α$^+$ T cells, CD4$^+$CD8α$^+$ blasts. Example shows rN + pustulan treated PBMCs.
