# Supplementary material for: Pustulan Activates Chicken Bone Marrow-Derived Dendritic Cells In Vitro and Promotes Ex Vivo CD4+ T Cell Recall Response to Infectious Bronchitis Virus
Source: Vaccines (Basel). 2020 May 15;8(2):226. doi: 10.3390/vaccines8020226 (PMC7349971; doi:10.3390/vaccines8020226)
Supplement: Supplementary file 1 [file vaccines-08-00226-s001.zip › vaccines-748283 sup table .pdf]

Supplementary table 1. Primers used for cytokine mRNA expression analysis in chicken bone marrow-derived dendritic cells

Target	Forward primer	Reverse primer	Accession number
IFN-γ	5′-GTGAAGAAGGTGAAAGATATCATGGA-3′	5′-GCTTTGCGCTGGATTCTCA-3′	NM_205149
TNF-α	5′-CGCTCAGAACGACGTCAA-3′	5′-GTCGTCCACACCAACGAG-3′	MF000729
IL-1β	5′-GCTCTACATGTCGTGTGTGATGAG-3′	5′-TGTCGATGTCCCGCATGA-3′	NM_204524
IL-6	5′-GCTTCGACGAGGAGAAATGC-3′	5′-GCCAGGTGCTTTGTGCTGTA-3′	NM_204628
CXCLi2	5′-GCCCTCCTCCTGGTTTCA-3′	5′-TGGCACCGCAGCTCATT-3′	NM_205498
IL-12p40	5′-AGATGCTGGCAACTACACCTG-3′	5′-CATTTGCCCATTGGAGTCTAC-3′	NM_213571
IL-18	5′-AGGTGAAATCTGGCAGTGGAAT-3′	5′-ACCTGGACGCTGAATGCAA-3′	NM_204608
HMBS	5′-GGTTGAGATGCTCCGTGAGTTT-3′	5′-GGCTCTTCTCCCCAATCTTAGAA-3′	XM_004947916
